# Talking to cancer patients about complementary therapies: is it the physician’s responsibility?

**DOI:** 10.3747/co.v15i0.279

**Published:** 2008-08

**Authors:** M.J. Verhoef, H.S. Boon, S.A. Page

**Affiliations:** * Department of Community Health Sciences, Faculty of Medicine, University of Calgary, Calgary, AB.; † Faculty of Pharmacy, University of Toronto, Toronto, ON.

**Keywords:** Complementary and alternative medicine, cam, physicians, patients, communication

## Abstract

**Background:**

To ensure the safety and effectiveness of cancer management, it is important for physicians treating cancer patients to know whether their patients are using complementary and alternative medicine (cam) and if so, why.

**Objective:**

Here, we discuss the ethical and legal obligations of physicians to discuss cam use in an oncology setting, and we provide practical advice on how patient–provider communication about cam can be improved.

**Results:**

Physicians have both ethical and legal obligations to their patients, including the obligation to respect patient autonomy. This latter obligation extends to use of cam by patients and needs to be addressed beginning early in the patient–provider relationship. Because lack of education in this field and lack of time during patient consultations are barriers to talking with patients about cam, we provide resources to facilitate such discussions. These resources include suggestions on how to discuss the topic of cam and a wide range of information sources.

**Conclusions:**

Discussing cam with patients is the physician’s responsibility, and such discussion will facilitate evidence-based, patient-centred cancer care.

## 1. INTRODUCTION

Studies report that most patients undergoing cancer treatment also choose to use selected forms of complementary and alternative medicine (cam), including natural health products 1,2. Reasons cited by cancer patients for cam use include treating the cancer, managing treatment side effects, enhancing quality of life and well-being, boosting the immune system, maintaining hope, and having more control over their cancer care 2–4.

It is important that physicians treating cancer patients know whether their patients are using cam and, if so, why. First, physicians need to know because of the possibility of direct adverse events associated with the use of cam. Second, interaction effects between conventional medicine and cam are possible, and the harms and benefits of cam may be misattributed to conventional treatment and thus may complicate treatment regimens. Third, patients may delay the use of conventional treatment when using cam. Finally, knowing why patients are using cam may provide important information about beliefs, values, expectations, and hopes on the part of the patient and will facilitate building a trusting relationship that will enhance the delivery of patient-centred cancer care.

However, research has shown that 40%–77% of patients who use cam therapies do not disclose their use of or interest in cam, or their desire to use cam, to their physicians because of concerns that the physicians will react negatively or will dismiss their questions 5–7. Patients may also think that physicians do not need to know that they are using cam, because the patients may believe that cam therapies are natural, completely safe, and not within the physicians’ scope of practice. Finally, patients often do not tell, simply because physicians do not ask about cam use.

In this paper, we discuss the ethical and legal obligations of physicians to discuss cam use in an oncology setting, and we provide practical advice on how patient–provider communication about cam can be improved.

## 2. DISCUSSION

### 2.1 Obligation of Physicians to Discuss CAM

Physicians have both ethical and legal obligations to their patients, including the obligation to respect patient autonomy. Operationally, respecting patient autonomy in the context of treatment decision-making means allowing patients to make choices 8. However, choice is meaningless if it is not made in light of all relevant information and advice 9. Although patients are the ones who must make the treatment decisions, physicians have the duty to inform patients about all therapeutic options, including cam. This means that physicians must be prepared to provide information and advice about

- benefits and likely outcomes of treatment,
- risks involved in treatment,
- possibility and probability of complications, and
- side effects and alternative treatment options.

This information is needed to meet the traditional ethical principles of non-maleficence (do no harm) and beneficence (offer a benefit) 10. Specifically for cam, the obligation to provide information and advice means that where risks are unknown and benefits are uncertain, it is necessary to highlight this absence of information.

Canadian courts have been very liberal and expansive in interpreting the disclosure obligations of physicians. In the context of informed consent, physicians are legally and ethically required to provide patients with detailed information about the evidence (for and against) the possible efficacy of treatments and to discuss costs, risks, and how a given treatment compares with other therapeutic options 11,12. The informed consent approach applies whether the recommended treatment is labelled biomedical or cam, and it raises the question of whether physicians must discuss cam treatment options to fulfil the informed consent requirement to compare a given treatment option with other therapeutic options.

It has been argued that an exploration of cam treatment options with patients is necessary, especially when information about cam options will be material—that is, significant—to a decision about a conventional treatment. Thus, if a patient’s decision about pursuing a particular biomedical treatment is likely to be influenced by knowing about specific cam treatments (including evidence of their safety, efficacy, and cost), then a physician has an obligation to include a discussion of these cam options as part of the informed consent process 12,13. Given the widespread use of cam among Canadians diagnosed with cancer, it appears reasonable to assume that cam options will be material to many patients. In addition, perhaps the only way to determine if cam options are relevant factors in the decision-making processes of patients is to open a dialogue about the issues.

There is increasing consensus in the literature about the importance of fully disclosing detailed information about the risks and benefits of cam interventions, including clear explanations of what is not known about them 11–13. The focus appears to be on protecting patients from physicians that promote cam products and therapies beyond what is believed by others to be supported by scientific evidence. Legally, physicians are required to practice in accordance with the “standard of care,” which generally refers to “the level of care the average and prudent health care professional in a given community would provide” 13. This standard changes over time and across cultural contexts. Although therapies with scientific evidence of safety and efficacy are unlikely to be judged outside the standard of care, scientific evidence is not the only criterion upon which such judgments are made.

In today’s culture of evidence-based practice, scientific evidence is becoming increasingly important 11, but clinical judgment and patient values are important, too 14. Thus, physicians who provide or recommend cam therapies for which there is little evidence could leave themselves open to charges of medical malpractice 11,13. In contrast, requirements to fully discuss cam options as “alternative” treatment options when recommending a conventional biomedical treatment may soon become standard practice.

Adams *et al.* 15 identified a wide range of patient-and physician-related factors that affect decision-making and subsequent use of cam, including

- severity of the illness,
- curability with conventional treatment,
- side effects of conventional treatment,
- quality of evidence of safety and efficacy of cam,
- degree of understanding of risks and benefits,
- knowing and voluntary acceptance of risks by the patient, and
- commitment to cam use by the patient.

Clearly, the need for physicians to assist patients in treatment decision-making is high and requires more than being informed about cam. Physicians also need to have effective (and non-judgmental) communication skills to manage the discussion. Patient–physician communication plays a crucial role, because these issues are best resolved by means of shared decision-making between patient and physician, where sufficient information is exchanged to create a consensus approach to deciding on the optimal clinical course.

### 2.2 How to Discuss CAM with Patients

It is becoming increasingly clear that patients have many legitimate needs and concerns that are not being met by conventional medicine. By adhering to the ethical criteria for informed decision-making and by honouring patient autonomy, physicians should be able to engage in open discussions with their patients about cam and to enable their patients to make sound decisions 6.

Not communicating with patients about cam may not only result in decreased trust within the therapeutic relationship, but also in selection by the patients of harmful, ineffective, and costly cam therapies. Patients who use cam may have unidentified needs or may be dissatisfied with the conventional care they are receiving. Once the issue of cam use is raised, the unidentified needs or dissatisfaction may come to the forefront and be addressed. However, the fact that relatively few physicians talk with their patients about cam suggests that these conversations are not easy for physicians. Lack of training in cam, limited training in communication skills, limited knowledge about cam, lack of scientific evidence about the risks and benefits of cam, and skepticism towards cam all appear to prevent physician engagement in such discussions.

Tasaki 16 found that patients identified these major barriers to successful discussions of cam:

- perceived indifference or opposition towards cam by the physician,
- emphasis on scientific evidence by the physician, and
- anticipation on the part of the patient of a negative response from the physician.

Initiating communication about cam is crucial. Table I summarizes a number of suggestions on how to encourage patients to talk about cam. Foley 19 underscores the need for such conversations by talking about “the need for us as oncology professionals to ‘Seek first to understand,’ to be open and to learn from our patients to serve them better.”

Eisenberg 20 was one of the first to propose a step-by-step strategy that conventionally trained physicians could use to proactively discuss cam use. This strategy involves a formal discussion of the treatment preferences and expectations of patients, the maintenance of symptom diaries, and follow-up visits to monitor for potentially harmful situations. In the absence of medical and legal guidelines, the proposed management plan emphasizes patient safety, the need for documentation in patient records, and the importance of shared decision-making. Although this strategy may be impractical and cumbersome in practice, Eisenberg should be credited for highlighting essential elements in physician–patient communication and for focusing on documentation and follow-up.

Informally, the five major steps to intervention—“ask, advise, assess, assist, and arrange” (5 A’s) 21—have been mentioned as important guidelines for communicating about cam, but a more focused approach has been presented by Cohen *et al.* 13. They suggest exploration (of the patient’s main issues), validation (acknowledge and commend the patient for seeking to resolve symptoms and improve health), empathy (with the patient’s desire to do everything possible), evaluation (consult with colleagues and other experts, and consult reputable sources of information), communication (share findings with the patient), and documentation of the conversations with the patient and of the patient’s progress as previously highlighted by Eisenberg 20.

Yet another perspective is provided by Frenkel *et al.* 22. These authors suggested that, to help cancer patients be truly informed and autonomous, physicians need to identify the patient’s beliefs, fears, hopes, and expectations; learn which conventional treatments have been tried, have failed, or have been rejected and why; make sure the patient understands the prognostic factors associated with his or her stage of disease, plus the potential benefits and harms of conventional medicine; acknowledge the patient’s spiritual and religious values and beliefs to understand how these affect health care choices; and assess the level of support that the patient has from friends, family, and community.

These frameworks approach the issue of communication with patients from slightly different angles, yet all are important, and physicians will most likely use elements of all approaches depending on the particular situation.

Implicitly, all models suggest that cam use should be inquired about from the beginning of contact with the patient, ideally before the patient starts using cam. This emphasis suggests that cam use should be made a regular part of history-taking. Physicians therefore need effective communication skills to fulfil a variety of roles, including collecting medical histories, answering patients’ questions, developing interpersonal relations, and suggesting treatment 16. Although this need seems obvious, it is yet another demand on physicians working in often busy and stressful situations.

Lastly, it is important to consider that, although disclosure of cam is essential, successful communication hinges on supporting patient autonomy even when the patient is making the decision to use a therapy of which a physician does not approve.

### 2.3 Current and Future Trends in Patient–Provider CAM Discussion

The importance of talking with patients about cam therapies is currently receiving much attention. Recently, the National Center for Complementary and Alternative Medicine (nccam) in the United States started the Time to Talk campaign 23, urging health care providers to talk about cam. The nccam Web site also includes tips on how to talk with patients. In addition, the *British Medical Journal* recently published a challenging editorial, “Wham, bam, thank you cam” 24, highlighting the need to discuss cam; however, the author’s question, “Alternative medicine is wildly popular ... but what are we supposed to do about it?” raises the challenge of finding relevant, evidence-based information.

Uncovering evidence-based information is especially difficult given the large number and heterogeneity of cam interventions. As a result, only a limited number of interventions have been adequately tested. Limited time in which to learn about cam and to discuss cam-related issues in patient consultations that are often already too short to address all patients’ concerns poses yet another challenge for physicians.

Currently, no single resource contains comprehensive summaries of the evidence base of all cam treatments relevant for patients diagnosed with cancer. In addition, the available evidence changes almost daily. However, helpful information can be found in a number of places. Table II includes a list of evidence-based cam resources that may help physicians when talking to patients about cam. Most are Web sites, because these are much easier to access (and are updated more regularly) than are books and articles. We recommend that physicians track the sources they access and find helpful, so that those sources are readily available when needed. Information on how to evaluate the wide range of cam information sources on the Web is available from nccam in the United States 25.

Because patients may see cam practitioners for cancer and cancer-related symptoms, it is also beneficial to be informed about the cam practitioners in the local area who are seeing cancer patients. The Prince of Wales Foundation for Integrated Health in the United Kingdom has published several guides for patients using cam 26. These guides include helpful information on finding cam practitioners and asking the right questions about those practitioners. An important aspect to assess is whether a given cam profession is regulated and whether a specific practitioner has adequate credentials.

It will be important in the future to ensure that cam is a topic in medical education, because all graduating physicians will encounter this issue in their practice. The Canadian cam in ume (undergraduate medical education) Web site provides useful resources for those involved in teaching medical students about cam 17. For physicians already in practice, continuing medical education may be a solution; however, most important is what can be learned from talking with patients: not only what they use, but also what their questions are.

## 3. CONCLUSIONS

Many people have already been using cam before a cancer diagnosis, and they consider it to be part of their health care. It is important to note that it is not possible to provide evidence-based, patient-centred care without engaging in a discussion of cam, including an exploration of the patient’s beliefs. Patients may be reluctant to discuss cam because of a fear of rejection or because of their beliefs about the complete safety of cam. It is therefore important that physicians initiate discussion of the topic—ideally, early in the relationship (for example, during initial history-taking), before patients have made any decisions about cam treatments. It truly is time to talk about cam with patients.

**TABLE I:** Communicating with patients: keeping the door open^17^,^18^

*What follows is a list of possible questions and issues that physicians can raise. Obviously, it is neither possible nor necessary to address all areas; however, even one good question may be the key to either opening the door to discussing cam or keeping the door open.*
1. Always ask about complementary and alternative medicine (cam) use—for example, “What else are you doing to take care of your cancer?” Ask in an open, non-judgmental way, and avoid using labels such as quackery, unscientific, and so on.
2. Watch for “non disclosing” clues: “You have read a lot about this. Have you seen other types of practitioners?”
3. Give permission for the patient to raise the topic by asking, “Many of my patients are interested in trying complementary therapies. Have you used any other therapies for this problem?”
4. Check with patients about their explanatory models: “What do you think is causing your symptoms [or cancer (because many patients have strong opinions of causes of cancer)]?”
5. Seek more information from patients and other sources: “Do you have any articles you can share with me?”
a. Be prepared for patients doing their own research.
b. Be aware of what they are being told about cam.
6. Explore why patients are using cam, and learn about their beliefs and values. It is important to consider that
a. a great deal more than evidence goes into a patient’s decision to use cam.
b. for many patients, care (enhancing well-being, easing suffering) is as important as cure.
7. Discuss the patient’s treatment preferences and expectations.
8. Review issues of efficiency and safety with respect to cam.
9. Be frank about your level of understanding or knowledge. It is okay not to know everything about cam.
10. Support the patient in efforts to obtain answers to important questions about risk and benefit. Ask yourself:
a. Is the cam therapy really dangerous?
b. Does it prohibit necessary medical care?
c. Can you work within the patient’s belief system to provide good care?
If the answer to the last question is yes, the next steps include negotiation and education. If the answer is no, the next step would be to arrive at a mutually acceptable course of action.
11. Discussing cam use does not mean that you are endorsing or promoting cam use.

**TABLE II:** Resource books and Web sites

*The Desktop Guide to Complementary and Alternative Medicine: An Evidence-based Approach*
Ernst E, Pittler MH, Wider B, editors.
2nd edition, 2006
480 pages, paperback
*Integrative Medicine: Principles for Practice*
(Chapter 23, pp. 535–549)
Kligler B, Lee R.2004
700 pages, hardcover
*Complementary and Alternative Medicine Secrets: Q&As about Integrating CAM Therapies into Clinical Practice*
(Chapters 54 and 55, pp. 363–388)
Kohatsu W.
2002
456 pages, paperback
*Integrative Medicine*
(Section 13, pp. 809–899; evidence for all treatments is rated)
Rakel D.
2nd edition, 2007
1238 pages, hardcover
*The Oxford Handbook of Complementary Medicine*
Ernst E, Pittler MH, Wider B, Boddy K.
2008
512 pages, paperback
CAMline
www.camline.ca
Center for Health and Healing
(a service of Beth Israel Medical Center in New York)
www.healthandhealingny.org
National Center for Complementary and Alternative Medicine (nccam)
nccam.nih.gov/health
Natural Medicines Comprehensive Database
www.naturaldatabase.com
Natural Medicines Comprehensive Database—Clinical Management Series
www.naturaldatabase.com/(S(st2arzb2hbi2v355rtipno2p))/nd/ClinicalMngt.aspx?cs=&s=ND
Natural Standard Database
www.naturalstandard.com
Turning Research into Practice (trip)
databasewww.tripdatabase.com
The University of Texas MD Anderson Cancer CenterComplementary/Integrative Medicine Education Resources
www.mdanderson.org/departments/cimer
Memorial Sloan–Kettering Cancer Center
www.mskcc.org/mskcc/html/44.cfm

## References

[1] Richardson MA, Sander T, Palmer JL, Greisinger A, Singletary SE (2000). Complementary/alternative medicine use in a comprehensive cancer center and the implications for oncology. J Clin Oncol.

[2] Sparber A, Bauer L, Curt G (2000). Use of complementary medicine by adult patients participating in cancer clinical trials. Oncol Nurs Forum.

[3] Boon H, Stewart M, Kennard MA (2000). The use of complementary and alternative medicine by breast cancer survivors in Ontario: prevalence and perceptions. J Clin Oncol.

[4] Verhoef M, Balneaves L, Boon H, Vroegindewey A (2005). Reasons for and characteristics associated with complementary and alternative medicine use among adult cancer patients: a systematic review. Integr Cancer Ther.

[5] Robinson A, McGrail MR (2004). Disclosure of cam use to medical practitioners: a review of qualitative and quantitative studies. Complement Ther Med.

[6] Pappas S, Perlman A (2002). Complementary and alternative medicine: the importance of doctor–patient communication. Med Clin North Am.

[7] Adler S, Fosket J (1999). Disclosing complementary and alternative medicine use in the medical encounter: a qualitative study in women with breast cancer. J Fam Pract.

[8] Weir M (2003). Obligation to advise of options for treatment—medical doctors and complementary and alternative medicine practitioners. J Law Med.

[9] Brophy E (2003). Does a doctor have a duty to provide information and advice about complementary and alternative medicine?. J Law Med.

[10] Cohen M (2006). Legal and ethical issues relating to use of complementary therapies in pediatric hematology/oncology. J Pediatr Hematol Oncol.

[11] Caulfield T, Feasby C (2001). Potions, promises and paradoxes: complementary medicine and alternative medicine and malpractice law in Canada. Health Law J.

[12] Ernst E, Cohen MH (2001). Informed consent in complementary and alternative medicine. Arch Intern Med.

[13] Cohen L, Cohen L, Kirkwood C, Russell N (2007). Discussing complementary therapies in an oncology setting. J Soc Integr Oncol.

[14] Sackett D, Strauss S, Richardson W, Rosenberg W, Haynes R (2000). Evidence-based Medicine. How to Practice and Teach EBM.

[15] Adams KE, Cohen MH, Eisenberg D, Jonsen AR (2002). Ethical considerations of complementary and alternative medical therapies in conventional medical settings. Ann Intern Med.

[16] Tasaki K, Maskarinec G, Shumay D, Tatsumura Y, Kakai H (2002). Communication between physicians and cancer patients about complementary and alternative medicine: exploring patients’ perspectives. Psychooncology.

[17] Community Health Sciences, University of Calgary (2003). Complementary and Alternative Medicine Issues in Undergraduate Medical Education [Web resource]. www.caminume.ca.

[18] Coulehan JL, Block MR (2006). The Medical Interview. Mastering Skills for Clinical Practice.

[19] Foley G (2002). The quest for understanding: a challenge in cancer care. Cancer Pract.

[20] Eisenberg DM (1997). Advising patients who seek alternative medical therapies. Ann Intern Med.

[21] Dosh SA, Holtrop J, Torres T, Arnold A, Baumann J, White L (2005). Changing organizational constructs into functional tools: an assessment of the 5 A’s in primary care practices. Ann Fam Med.

[22] Frenkel M, Ben-Arye E, Baldwin C, Sierpina V (2005). Approach to communicating with patients about the use of nutritional supplements in cancer care. South Med J.

[23] National Institutes of Health, National Centre for Complementary and Alternative Medicine (nccam) Time to Talk [Web page]. nccam.nih.gov/timetotalk.

[24] Kamerow D (2007). Wham, bam, thank you cam. BMJ.

[25] National Institutes of Health, National Centre for Complementary and Alternative Medicine (nccam) 10 Things to Know About Evaluating Medical Resources on the Web [Web page]. nccam.nih.gov/health/webresources.

[26] Pinder M, Pedro L, Theodorou G, Treacy K, Miller W (2005). Complementary Healthcare: A Guide for Patients.

